# Colorectal Cancer and Polyps in Diverticulosis Patients: A 10-Year Retrospective Study in 13680 Patients

**DOI:** 10.1155/2019/2507848

**Published:** 2019-12-01

**Authors:** Fadi Abu Baker, Jesus Alonso Z'cruz De La Garza, Amir Mari, Abdel-Rauf Zeina, Amani Bishara, Oren Gal, Yael Kopelman

**Affiliations:** ^1^Department of Gastroenterology and Hepatology, Hillel Yaffe Medical Center, Affiliated to the Technion Faculty of Medicine, Hadera, Haifa, Israel; ^2^Department of Surgery, Hillel Yaffe Medical Center, Affiliated to the Technion Faculty of Medicine, Hadera, Haifa, Israel; ^3^Department of Gastroenterology, Nazareth EMMS Hospital, Affiliated to the Faculty of Medicine, Bar Ilan University, Israel; ^4^Department of Radiology, Hillel Yaffe Medical Center, Affiliated to the Faculty of Medicine, Technion-Israel Institute of Technology, Haifa, Israel

## Abstract

**Introduction:**

Shared by certain epidemiological and etiological characteristics, diverticulosis and colorectal cancer (CRC) as well as colonic polyps has long been linked. This association was studied in several heterogeneous studies but has reported inconsistent results. Clarifying the association is clinically relevant for endoscopist awareness and potential modification of screening and surveillance intervals for diverticulosis patients.

**Methods:**

In this retrospective single-center study, patients diagnosed with diverticulosis on colonoscopy over a 10-year period were included. Each diverticulosis patient was matched with 1 control by age, gender, setting (inpatient/outpatient), and procedure's indication. CRC and polyp detection rates were recorded and compared between the groups before and after adjustment for bowel preparation quality and exam completion. CRC location was recorded and compared between groups.

**Results:**

A cohort of 13680 patients (6840 patients with diverticulosis and 6840 matched controls) was included. Diverticulosis was located mainly to the sigmoid and left colon (94.4%). The CRC diagnosis rate was lower in the diverticulosis group (2% vs. 4.5%, odds ratio = 0.472, *P* < 0.001, and 95%CI = 0.382‐0.584). Moreover, location of CRC was unrelated to diverticulosis location, as more CRCs in the diverticulosis group were located proximal to the splenic flexure as compared to the control group (42.5% vs 29.5%, respectively; *P* = 0.007). Diverticulosis, however, was associated with an increased polyp detection rate compared to controls (30.5% vs. 25.5%; odds ratio = 1.2, *P* < 0.001, and 95%CI = 1.11‐1.299).

**Conclusion:**

We demonstrated that diverticulosis was not associated with an increased risk for CRC. A possible increased polyp detection rate, however, warrants further evaluation in large prospective studies.

## 1. Background

Diverticulosis is considered as one of the most common and burdensome GI disorders [1, 2]. The underlying pathological mechanisms resulting in diverticular formation of a colonic wall is still largely unknown. Diverticula develop at well-defined points of weakness in the circular muscle of colon and are likely to be the result of complex interactions between environmental and heritable factors including diet, increased age, and decreased colonic motility, among others [3, 4]. Typically, diverticulosis is identified incidentally at colonoscopy or imaging studies performed for various indications, and the majority of patients with diverticulosis remain asymptomatic throughout their lifetime [5].

Several observations hold that certain epidemiological and etiological characteristics are shared between colonic diverticulosis and colorectal cancer, suggesting a possible association between these two conditions. The prevalence of these conditions is markedly on the rise in the last decades, and they both are detected frequently in aged people as well as western population and industrialized countries [6–8]. Western diet, namely, low dietary fiber and high total fat, has been largely regarded to play a major role in the pathogenesis and was epidemiologically associated with an increased risk of both conditions [9–13].

The abovementioned connection is of great clinical relevance, as several reports demonstrated that patients with diverticular disease have a higher risk of harboring colonic cancer [14–16]. Above and beyond, one meta-analysis demonstrated that diverticular disease was associated as well with increased detection of colorectal adenomas [17].

However, data are still controversial and inconclusive as other recent studies failed to confirm this association [18–20]. Moreover, the vast majority of the studies inspecting a possible link between both conditions were limited by small patient numbers and did not account for multiple confounders that are known to affect CRC and polyp detection such as patients' demographics, procedure's indication, setting, quality of bowel preparation, and exam completion.

Taken together, unraveling the dilemma and clarifying the association between both conditions appear to be clinically relevant, as modifying screening or surveillance intervals for CRC and polyp follow-up may be warranted accordingly in patients with diverticular disease.

The present study is aimed at comparing the CRC diagnosis rate and location as well as polyp detection rate between patients with diverticular disease and a matched group without diverticulosis with adjustment for abovementioned confounders.

## 2. Methods and Settings

We conducted a retrospective, large cohort study, which examined consecutive patients who underwent colonoscopies over a 10-year period within the gastroenterology department at the Hillel Yaffe Medical Center, a university-affiliated hospital in Israel. All patients' data were collected from our department's electronic record system. We searched endoscopy reports to identify all patients with a diagnosis of diverticulosis to create a study group. For a control group, each patient from the study group was matched with 1 control patient by age, gender, setting (inpatient/outpatient), and procedure's indication. Patients were excluded if they were less than 18 years, had prior diagnosis of colon cancer, or if full data set is missing. Endoscopy findings including cancer diagnosis and location as well as polyp detection were recorded in both groups. Diverticulosis location was documented as well in the diverticulosis group. Whenever an endoscopic diagnosis of colorectal cancer was encountered, histology reports were reviewed to confirm diagnosis.

We compared the rate of CRC and polyp diagnosis between both groups and use multivariable analysis to adjust for adequacy of bowel preparation (adequate/inadequate) and depth of examination (cecal intubation confirmed or not), in order to identify independent association of diverticulosis with CRC and polyp detection. CRC location was documented according to endoscopy reports, and we compared its location between both groups. Diverticulosis and CRC location was classified as proximal (proximal to splenic flexure) or distal (splenic flexure or distal). The local institutional Helsinki ethics board approved the study and granted exemption from informed consent in this retrospective study as patients were receiving standard care without relation to the study.

## 3. Statistical Analysis

This statistical analysis is dealing with cohort of “big data” (40128 patients), of them 6840 patients with diverticulosis (study group). We used the Propensity Score Matching in R program version 3.3 to divide the total cohort to 1 : 1 ratio (study and control group). Descriptive statistics in terms of mean, SD, and percentiles were preformed to the whole parameters in the study. Differences between the two groups (diverticulosis diagnosed vs. matched group) in the quantitative parameters were demonstrated by *t*-test. For the categorical parameters, we used fisher exact tests. Multivariate logistic regression model was used to determine the effect of the independent parameters associated with CRC. SPSS version 25 was also used for statistical analysis. *P* < 0.05 was considered as significant.

## 4. Results

We included a large cohort of 40128 patients who underwent colonoscopy at our hospital. We searched endoscopy reports and identified 6840 patients (17%) with diverticulosis diagnosed during the study period. A matched group of 6840 control patients (at 1 : 1 ratio) was included for final analysis. Baseline characteristics of both groups were similar and are provided in Table 1. The overall mean age was 68.3 ± 11.0 years (range: 18-101), with a slight male predominance (52.1%). The vast majority of the procedures (78.4%) were performed in the outpatient setting. Procedures' indications did not differ significantly between groups. The most common indications for colonoscopy were abdominal pain and diarrhea (21.1%), rectal bleeding (14.2%), and anemia (13.2%).

The CRC diagnosis rate was lower in the diverticulosis group (2% vs. 4.5%; *P* < 0.01) while the polyp detection rate was surprisingly higher (30.5% vs. 25.5%; *P* < 0.01) as compared to the matched group (Table 2). Cecal intubation rate (92.8% vs. 84.1%; *P* < 0.01), adequate bowel preparation rate (90.7% vs. 84.1%; *P* < 0.01), and terminal ileum intubation rate (2.2% vs. 1.6%; OR = 1.4 and 95%CI = 1.096‐1.805; *P* = 0.008) were significantly higher in the diverticulosis group (Table 2). Multivariate analysis (Table 3) to account for these variables revealed similar trends as diverticulosis patients were associated with less CRC diagnosis (odds ratio = 0.472, *P* < 0.001, and 95%CI = 0.382‐0.584) but increased polyp detection rate (odds ratio = 1.2, *P* < 0.001, and 95%CI = 1.11‐1.299).

Diverticulosis and CRC locations are demonstrated in Table 4. Diverticulosis was located mainly to the distal colon (94.4%). Location of CRC was unrelated to diverticulosis location, as more CRCs in the diverticulosis group were located proximally compared to the control group (42.5% vs. 29.5%, respectively; *P* = 0.07).

## 5. Discussion

The current study was designed to clarify several aspects of diverticulosis and its possible association with CRC and polyp diagnosis. This association has long been studied in observational, cross-sectional, and case-control studies and has reported inconsistent results. Moreover, small patient numbers and heterogeneous study design contributed to conflicting conclusions. In the current study, we included a large cohort over a 10-year period and performed group matching followed with multivariate analysis in order to account for as many confounders as possible that may have influenced results of preceding studies.

We found that diverticulosis patients were not associated with an increased rate of CRC diagnosis compared to a matched group (2% vs. 4.5%; OR = 0.472, 95%CI = 0.382‐0.584, *P* < 0.001). Our findings confirm the findings from other recent studies that diverticulosis is not associated with increased CRC diagnosis. A nationwide case-control study found that diverticular disease does not increase the risk of colon cancer in the long term, and a history of diverticular disease does not affect colon cancer mortality [18]. Our findings are also in concordance with a study by Meurs-Szojda et al. on more than 4200 colonoscopies which demonstrated a negative correlation between colon cancer and diverticulosis [21].

Moreover, we provided detailed location of diverticulosis and colon cancer in our cohort. Similar to different reports in western population [6, 7, 9], diverticulosis was located predominantly to the distal colon as more than 94% of diverticula were located to sigmoid and descending colon. In this regard, not only we demonstrated that CRC was located into sigmoid and descending colon in less than 35% but also we showed that more CRCs in the diverticulosis group were located proximally compared to the control group (Table 3) (42.5% vs. 29.5%, respectively; *P* = 0.07). Consistent findings were reported by Cooper et al. who showed that diverticulosis associated interval cancers were somewhat more likely to be in the proximal colon and less likely to be in the distal colon [15]. Taken together, these findings reinforce the conclusion that CRC and diverticulosis are unrelated.

One worth mentioning finding in this study, however, is the increased polyp detection rate in diverticulosis patients (30.5% vs. 25.5%; OR = 1.2, 95%CI = 1.11‐1.299, *P* < 0.01). This observation is supported by several other studies reporting that patients with diverticulosis have a higher risk of colorectal polyps as compared to those without [22, 23]. One meta-analysis found a significant 1.67-fold increased odds of developing adenomas in patients with diverticulosis [17]. Unfortunately, we were unable to determine the location and histologic type of these polyps in the current study. However, given the lower CRC diagnosis rate in the diverticulosis patients, this may point out that the majority of the detected polyps were of low dysplastic progression potentials (diminutive/hyperplastic polyps), thus explaining the low CRC diagnosis albeit the high PDR. Nevertheless, this observation needs further validation by a large prospective cohort study.

Furthermore, we demonstrated that diverticulosis has no significant effect on the outcome of colonoscopy. Linked with suboptimal bowel preparation, it is thought that diverticulosis may cause technical difficulty to perform a complete colonoscopy as a result of a spastic colon and luminal narrowing [24–26]. However, we demonstrated the contrary as quality indicators such as the cecal intubation rate (92.8% vs. 84.1%; OR = 2.45 and 95%CI = 2.187 − 2.738; *P* < 0.01) and the adequate bowel preparation rate (90.7% vs. 84.1%; OR = 1.853 and 95%CI = 1.670‐2.057; *P* < 0.01) as well as the terminal ileum intubation rate (2.2% vs. 1.6%; OR = 1.4 and 95%CI = 1.096‐1.805; *P* = 0.008) which were even better in diverticulosis patients compared to those without. Similar findings were reported by Gohil et al. who found that diverticulosis did not adversely affect the cecal intubation rate, withdrawal times, or sedation requirements [27].

One of the strengths of the current study includes the large number of participants involved as well as the inclusion of multiple factors such as procedures' settings and indications reflecting real daily practice. Our study has limits inherent in its retrospective nature. Besides, other possible factors that may have affected endoscopy findings such as withdrawal time and variable endoscopist experience could not be obtained and were not included.

In conclusion, diverticulosis apparently is not linked with an increased risk of CRC but is possibly associated with an increased polyp detection rate. Prospective studies to clarify these findings are warranted.

## Figures and Tables

**Table 1:** Baseline characteristics of diverticulosis and matched groups.

Characteristics	Matched group (*N* = 6840)	Diverticulosis group (*N* = 6840)	*P* value
Age at test (years)	68.3 ± 11.0	68.3 ± 11.0	*P* = 0.90
Gender (male)	3570 (52.2%)	3566 (52.1%)	*P* = 0.93
Setting (outpatient)	5365 (78.5%)	5347 (78.2%)	*P* = 0.69
Procedures' indication			
Personal history of polyps	657 (9.6%)	641 (9.4%)	*P* = 0.64
Abdominal pain/diarrhea	1432 (20.9%)	1454 (21.3%)	*P* = 0.66
IBD follow-up	41 (0.6%)	44 (0.6%)	*P* = 0.83
Past colonic surgery	189 (2.8%)	187 (2.7%)	*P* = 0.92
Anemia	876 (12.8%)	922 (13.5%)	*P* = 0.26
Positive FOBT	497 (7.3%)	521 (7.6%)	*P* = 0.45
Rectal bleed	947 (13.8%)	994 (14.5%)	*P* = 0.26
Family history of CRC	400 (5.8%)	416 (6.1%)	*P* = 0.59
Screening	248 (3.6%)	254 (3.7%)	*P* = 0.82
Constipation	747 (10.9%)	777 (11.4%)	*P* = 0.43
Imaging findings	394 (5.8%)	382 (5.6%)	*P* = 0.66
Weight loss	185 (2.7%)	237 (3.5%)	*P* = 0.12

**Table 2:** Endoscopic findings in both groups.

Endoscopic findings	Matched group (*N* = 6840)	Diverticulosis group (*N* = 6840)	*P* value
Polyp detection rate	1747 (25.5%)	2083 (30.5%)	*P* < 0.001
CRC diagnosis rate	306 (4.5%)	140 (2.0%)	*P* < 0.001
Complete procedure	5750 (84.1%)	6349 (92.8%)	*P* < 0.001
Adequate bowel preparation	5748 (84.1%)	6205 (90.7%)	*P* < 0.001
Terminal ileum intubation	108 (1.6%)	151 (2.2%)	*P* = 0.008

**Table 3:** Risk factors for colorectal cancer diagnosis, a multivariate analysis.

Characteristics	*P* value	Odds ratio	95% CI	
			Upper	Lower
Age at test (years)	<0.01	1.042	1.031	1.052
Gender (male)	0.357	1.099	0.899	1.344
Group (diverticulosis)	<0.01	0.472	0.382	0.584
Incomplete exam	<0.01	0.294	0.232	0.373
Procedures' indication				
Personal history of polyps	0.102	1.478	0.925	2.362
Abdominal pain/diarrhea	0.954	0.990	0.703	1.393
IBD follow-up	0.300	1.138	0.892	1.452
Past colonic surgery	0.299	0.612	0.242	1.546
Anemia	<0.01	2.912	2.168	3.911
Positive FOBT	<0.01	2.830	1.902	4.210
Rectal bleed	<0.01	3.504	2.556	4.803
Family history of CRC	0.506	1.066	0.883	1.287
Screening	0.350	0.572	0.177	1.845
Constipation	0.077	0.672	0.433	1.043
Imaging findings	<0.01	8.594	6.250	11.817
Weight loss	0.269	1.359	0.789	2.340

**Table 4:** Diverticulosis and colorectal cancer location in both groups.

Location		Diverticulosis group		Matched group
		CRC	Diverticulosis^∗^	
Distal colon	Rectum	0%	20%	23.90%
	Rectosigmoid	2.6%	7.60%	14%
	Sigmoid	84.1%^∗^	16.70%	17.30%
	Descending colon	23%^∗∗^	11%	14%

Proximal colon	Transverse colon	2.6%	4.90%	3.70%
	Ascending colon	5%^∗∗∗^	27.80%	17.90%
	Cecum	3%^∗∗∗∗^	9.80%	7.30%
	Undetermined	0%	2.20%	2%

^∗^Sigmoid (alone: 65%, sigmoid+descending: 12.5%, sigmoid+transverse: 2.6%, sigmoid+ ascending/cecum: 4%). ^∗∗^Descending colon (alone: 10.5%, sigmoid+descending: 12.5%). ^∗∗∗^Ascending colon (alone: 2%, sigmoid+ascending: 3%). ^∗∗∗∗^Cecum (alone 1%, sigmoid+cecum: 2%).

## Data Availability

The datasets used and/or analyzed during the current study are available from the corresponding author on reasonable request.
